# The effectiveness of combined bracing and exercise in adolescent idiopathic scoliosis based on SRS and SOSORT criteria: a prospective study

**DOI:** 10.1186/1471-2474-15-263

**Published:** 2014-08-06

**Authors:** Stefano Negrini, Sabrina Donzelli, Monia Lusini, Salvatore Minnella, Fabio Zaina

**Affiliations:** 1 Department of Clinical and Experimental Sciences, University of Brescia, Viale Europa 11 Brescia, Italy; 2 IRCCS Don Gnocchi Foundation, Milan, Italy; 3 ISICO (Italian Scientific Spine Institute), Milan, Italy

**Keywords:** Adolescent Idiopathic Scoliosis, Bracing, Exercises

## Abstract

**Background:**

Recently an RCT confirmed brace efficacy in adolescent idiopathic scoliosis (AIS) patients. Previously, a Cochrane review suggested also producing studies according to the Scoliosis Research Society (SRS) criteria on the effectiveness of bracing for AIS. Even if the SRS criteria propose a prospective design, until now only one out of 6 published studies was prospective. Our purpose was to evaluate the effects of bracing plus exercises following the SRS and the international Society on Scoliosis Orthopedic and Rehabilitation Treatment (SOSORT) criteria for AIS conservative treatment.

**Methods:**

Study design/setting: prospective cohort study nested in a clinical database of all outpatients of a clinic specialized in scoliosis conservative treatment.

Patient sample: seventy-three patients (60 females), age 12 years 10 months ±17 months, 34.4±4.4 Cobb degrees, who satisfied SRS criteria were included out of 3,883 patients at first evaluation.

Outcome measures: Cobb angle at the end of treatment according to SRS criteria : (unchanged; worsened 6° or more, over 45° and surgically treated, and rate of improvement of 6° or more).

Braces were prescribed for 18–23 hours/day according to curves magnitude and actual international guidelines. Weaning was gradual after Risser 3. All patients performed exercises and were managed according to SOSORT criteria. Results in all patients were analyzed according to intent-to-treat at the end of the treatment. Funding and Conflict of Interest: no.

**Results:**

Overall 34 patients (52.3%) improved. Seven patients (9.6%) worsened, of which 1 patient progressed beyond 45° and was fused. Referred compliance was assessed during a mean period of 3 years 4 months ±20 months; the median adherence was 99.1% (range 22.2-109.2%). Employing intent-to-treat analysis, there were failures in 11 patients (15.1%). At start, these patients had statistically significant low BMI and kyphosis, high thoracic rotation and higher Cobb angles. Drop-outs showed reduced compliance and years of treatment; their average scoliosis at discontinuation was low: 22.7° (range 16-35°) at Risser 1.3 ± 1.

**Conclusions:**

Bracing in patients with AIS who satisfy SRS criteria is effective. Combining bracing with exercise according to SOSORT criteria shows better results than the current literature.

## Background

Recently a multicenter RCT investigating the role of bracing in AIS patients at risk of curve progression, confirmed brace efficacy. The authors conclude that bracing significantly decreased the progression of high risk curves and that the longer the brace wear the best were the results [1].

Previously, a Cochrane review [2] favoured bracing in adolescent idiopathic scoliosis (AIS) treatment. The evidence was however based on a “very low quality” prospective observational cohort that found bracing to be more effective in reducing curve progression to surgery compared with observations only and electrical stimulation [3]. To increase this quality we would need randomized controlled trials (RCTs): nevertheless, RCTs either the last one by Weinstein [1], as the previously published, in the Netherlands [4-6], and in the US [7] failed, and had to be changed to observational [8], due to the difficulty in recruiting patients. Recently, results of an RCT on the SpineCor brace have been presented at the SOSORT Meeting (Coillard et al. [9]).

In 1995 the SRS proposed the methodological criteria for studies on brace effectiveness [10]. For inclusion: at least age 10 at brace prescription; Risser 0–2; primary curve angles between 25° and 40°; no previous treatment; if female, either premenarchal or less than 1 year postmenarchal. For outcome assessment: curve progression less than 6° or more than 5°; curves exceeding 45 degrees at maturity; and surgery recommended/undertaken. For the last criterion, a 2-year follow-up beyond maturity is required. Design should be prospective, and an intent to treat analysis should be performed, including all patients.

In 2008, the international Society On Scoliosis Orthopedic and Rehabilitation Treatment (SOSORT) proposed the criteria for management of braced patients in clinics and during research studies [11]. The SOSORT criteria include 14 recommendations, grouped in 6 Domains (Experience/competence, Behaviours, Prescription, Construction, Brace Check, Follow-up). They mainly stress the importance of a good and expert conservative team surrounding the patient and family, to serve as a guarantee of quality of braces and increase compliance, that is a main determinant of final results [12-15].

In the already mentioned Cochrane review [2] it is suggested that, while waiting for RCTs results, studies according to the SRS (and SOSORT) criteria are tools to obtain evidence on the effectiveness of bracing for AIS. Today it is possible to find 5 studies respecting all these criteria [9,16-19]: looking at them as a whole, bracing seems to alter positively the natural history of AIS, and apparently best results can be achieved when the SOSORT criteria are satisfied [2].

Nevertheless, even if the SRS criteria propose to follow a prospective design, until now only one out of the 6 published studies respecting the SRS criteria was prospective [20]: this did not respect the SOSORT criteria. Such a design gives stronger evidence, since it allows to perform not only an efficacy analysis (results on patients who completed treatment), but also an intent-to-treat analysis (results on all treated patients) [10].

Aim of the present study was to evaluate the effects of a complete conservative treatment (bracing plus exercises) strictly following the SRS and SOSORT criteria.

## Methods

### Study design

We performed a cohort prospective study nested in a clinical database including all patients referred to our Institute (an outpatient clinic specialized in idiopathic scoliosis clinical evaluation and treatment, with patients coming from all over Country and abroad).

The prospective database from which data were extracted was started in March 2003, the target population was made by AIS patients at their first evaluation recruited into the database during the period comprised between march 2003 and the 31^st^ of July 2008. The study has been performed on the 31^st^ of December 2011.

All patients gave an informed consent to their data management for clinical and research purposes. Since this study is based on the regular everyday clinical activity of our Institute, an Ethical Committee approval was not required.

### Population

**Figure 1:**
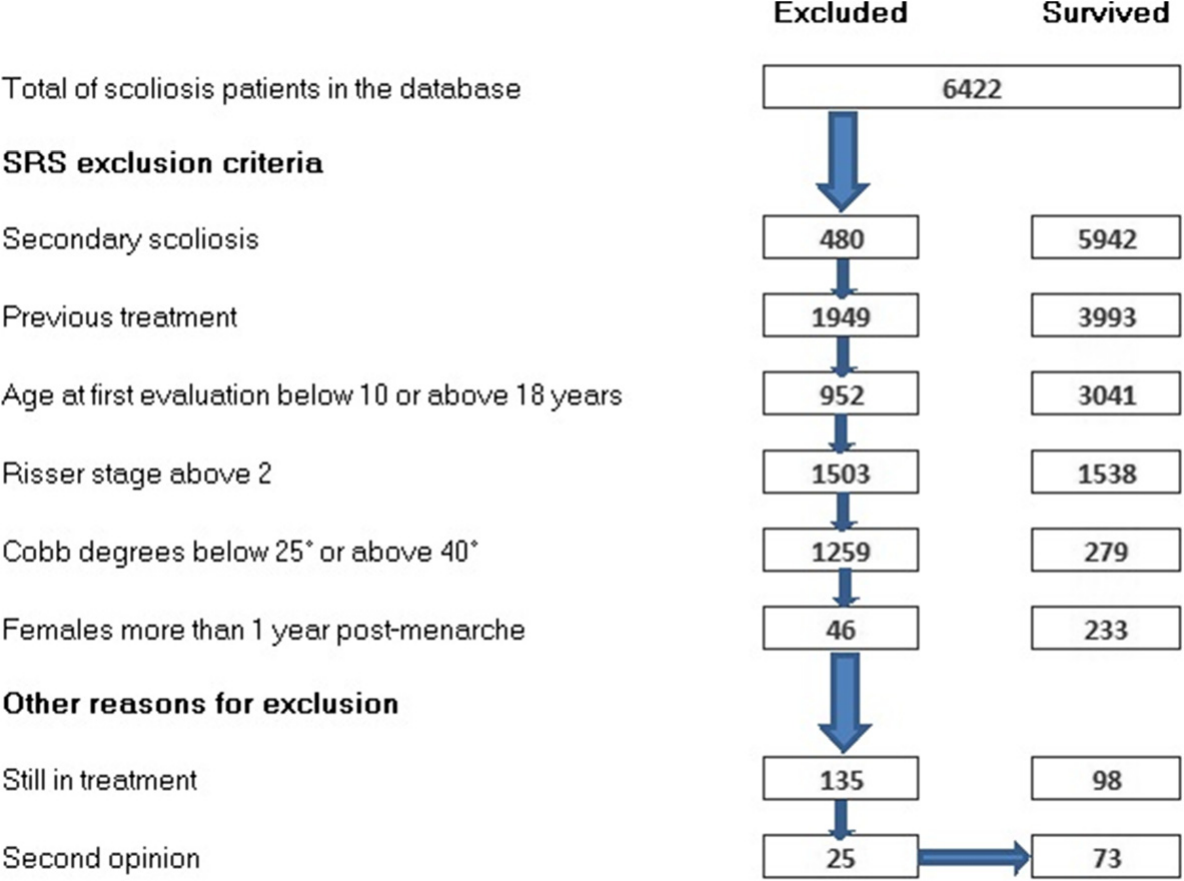
Flow chart of the selection of the study from the entire sample of patients included in the clinical database.

We strictly followed the SRS inclusion criteria [10]. The process of extraction is synthesized in Figure 1, in which the number of patients progressively excluded are listed. We evaluated 3,883 patients; at start of the study, of which 148 patients respected the SRS criteria. Thirty-nine adolescents were excluded because they only came, to our institution, for second opinion consultation, and were not treated by us. Additionally, 36 patients were excluded because, at the end of the study, had not yet completed their treatments.

Finally, 73 patients (60 females - 82.2% - and 13 males - 17.8%) have been included. Average curves were 30.4 ± 4.4, with an average age of 12.10 ± 1.05 years. Thirty-nine (53.4%) had single, 32 (43.8%) double and 2 (2.7%) had triple curves (Table 1). For the purposes of analysis, patients were also classified in terms of convexity of curves: typical curves being right thoracic, left thoracolumbar/lumbar curves, and atypical ones being the opposite convexities (Table 1).

**Table 1:** Topographic classification of patients included in the study

**Type**	**%**	**Localization**	**%**	**Convexity**	**%**	**Type of curve**
Single	53.42%	Thoracic	16.44%	Right	15.07%	Typical
				Left	1.37%	Atypical
		Thoraco-lumbar	21.92%	Right	6.85%	Atypical
				left	15.()7%	Atypical
		Lumbar	l5.07	Right	0.00%	Atypical
				left	15.07%	Typical
Double	43.84%	Thoracic-Lumbar	20.55%	Right-Left	19.18%	Typical
				Left-Right	1.37%	Atypical
		Thoracic Thoracolumbar	17.81%	Right Left	12.33%	Typical
				Left-Right	5.48%	Atypical
		Double thoracic (proximal-distal)	5.48%	Right-Left	1.37%	Atypical
				Left-Right	4.11%	Typical
Triple	2.74%	Double Thoracic-Thoracolumbar	2.74%	Left-Right-Left	2.74%	Typical
				Right Left Right	0.00%	Atypical

For analysis’ purposes, patients were also classified in terms of typical and atypical curves, the first ones including thoracic right, thoracolumbar/lumbar left and Moe type, the second ones for the opposite convexities.

### Methods

All patients were proposed to be treated with braces and exercises. Braces were personalized by physicians according to individual needs:

• The Sibilla brace [21] was preferred in case of low rigidity and BMI, and reduced Cobb degrees: it was prescribed in 61.6% of included patients

• The Lyon [22] (before 2004–5) and Sforzesco [21] braces (from 2005) were preferred in cases of high rigidity and BMI, and severe Cobb degrees (over 35°): they were prescribed in 16.4% and 13.7% of cases respectively

• The SpineCor brace [23] was prescribed after 2008, only in cases below 30° and low rigidity: it was used in 6.8% of cases.

At start of treatment, hours of brace use were personalized, and patients were grouped accordingly:

• Full time (22–23 hours per day – h/d): 30 patients,

• Part-time (16–18 h/d): 21 patients,

• Intermediate (19–21 h/d): 22 patients.

Prescriptions were in agreement with international guidelines of AIS treatment and all the physiscian involved in the study were part of a team sharing all the treatment aim and therapeutical choices.

During the period of the study, the Thermobrace sensor was not used to measure the exact compliance, but we assumed similar results as those previously published [24].

The main rule observed during weaning was to avoid reducing hours of treatment below 18 h/d before reaching Risser 3 stage. According to this rule, a 2 hours reduction every 6 months started since first follow-up visit (usually performed after 4 months), with two exceptions: worsening (no reduction) and first control visit in case of full time wearing (one hour reduction only). The combination of the two rules, drive to final weaning on average 2.5 years after Risser 3, generally corresponding to Risser 5. As already described [25], this gradual weaning is performed to allow an adaptation of the postural system that, combined with exercises performance, should increase the possibility of stabilization of best results obtained.

All patients were prescribed Physiotherapic Specific Exercises (PSE) [15]: 3 patients did not perform any exercise (NE), 35 followed usual physiotherapy (UP) and 35 came to our Institute to learn PSE according to the SEAS school [26], that proved to be able to reduce correction loss during brace weaning [27], and to increase correction at first brace wearing [28].

SOSORT management criteria were fully respected (43/44 criteria fulfilled, with one not applicable) [11].

### Outcome criteria

End of treatment was defined as follows: indication by the treating physician and/or achievement of Risser 3 stage according to the European staging, corresponding to Risser 4 in the US [29,30].

The SRS outcome criteria were followed [10]. These included percentage of patient unchanged (less than 6° progression); worsened (6° or more); over 45° at the end of treatment; surgically treated. Since scoliosis can also be improved [25,31], we also added rate of improvement (defined as a reduction of 6° or more). Moreover, radiographic and clinical data have been computed [25,32].

### Statistical analysis

Conforming the SRS criteria [10], we performed an Intent-to-treat analysis, where failures included patients that:

• Reached 45° at the end-of-treatment;

• Were fused;

• Dropped-out (treatment stopped before reaching end-of-treatment) without any final radiographic evaluation;

• Dropped-out at Risser stage 0 or 1;

• Dropped-out at European Risser 2 (corresponding to US Risser 3) [29,30], only if the curve was of 35° Cobb or more: in fact, at this stage the risk of fusion and/or progression to 45° or more is negligible [10].

An Intent-to-treat analysis gives the overall usefulness of a treatment, but in many cases (such as that of braces) it is interesting also to know which results can be achieved in case patients decide to adhere to treatment: for this reason we performed also an efficacy analysis, looking only at patients who reached the end-of-treatment.

## Results

Looking at the overall results (Table 2), 7 (9.6%) patients worsened, 1 (1.4%) progressed beyond 45° and was the only one fused; 8 patients (11%) dropped-out from the study before reaching the end-of-treatment.

**Table 2:** Results according to the two main analysis performed

	**Total population**		**Improved**			**Unchanged**			**Worsened**			**Over 450**			**Failures (surgery)**		
	**N**	**%**	**N**	**%**	**C195**	**N**	**%**	**C195**	**N**	**%**	**C195**	**N**	**%**	**C195**	**N**	**%**	**CI95**
Intent-to- treat analysis	73	100%	34	46.5%	34.3-56.6%	27	36.9%	25.6-47.1%	7	9.6%	4.8-18.5%	1	1.4%	0.3-7.3%	4	5.5%	4.2-15.0%
Efficacy analysis	65	100%	33	50.8%	40.2-64.2%	26	40.0%	29.9-53.6%	4	6.2%	2.6-15.2%	1	1.5%	0.4-8.4%	1	1.5%	0.4-8.4%

Drop-outs (Table 3) concluded treatment after 17 ± 7 months of treatment. In this group, four patients did not have a final x-ray, and consequently it was not possible to know if they had reached Risser 3 stage (end-of-treatment). Patients dropped-out who had final x-rays had on average 22.7° (range 16-34°) of scoliosis with a Risser stage 0 or 1 in 1 case each, and 2 in 4 cases.

**Table 3:** Charactheristic of the patients that finished the study before end of treatment (physician prescription and/or Risser 3 stage) (drop-outs)

**Patient**	**Cobb degrees**	**Risser stage**	**Final result**
1	35	2	Failure
2	34	0	Failure
3	28	2	Success
4	24	2	Success
5	20	2	Success
6	20	0	Failure
7	20	2	Success
8	16	2	Success

Definition of failure or success according to what reported in the text.

According to the Intent-to-treat analysis (Table 2), failures were 11 (15.1%); in terms of Efficacy analysis, out of the 63 patients who reached the end-of-treatment, 3 patients progressed (4.8%), 1 reached 45° and was fused (1.6%). All subgroup analysis suggested by the SRS Committee [10] are listed in Table 4.

**Table 4:** Subgroup analysis suggested by the SRS Committee

		**Total population**	**Improved**	**Unchanged**	**Worsened**	**Over 45**	**Surgery**
Number of major curves	Single	38	22	14	2	0	0
	Double major	32	13	15	2	1	1
	Triplemajor	2	1	1	0	0	0
Topographic classification	Thoracic	12	5	6	1	0	0
	Thoracolumbar	16	12	4	0	0	0
	Lumbar	10	5	4	l	0	0
	Thoracic-Lumbar	16	6	7	1	1	1
	Thracic-Thoracolumbar	13	6	6	1	0	0
	Double thoracic	3	1	2	0	0	0
	Double thoracic- lumbar	2	1	1	0	0	0
Curvature type	Typical	61	32	23	4	1	1
	Atypical	11	4	7	0	0	0
Magnitude of curvature	25-30	40	21	18	l	0	0
	31-35	20	8	10	2	0	0
	36-40	12	7	2	1	1	1
Skeletal maturity	Risser 0	48	23	21	2	1	1
	Risser 1	10	5	3	2	0	0
	Risser 2	14	8	6	0	0	0
Brace	Sibilla	45	27	17	1	0	0
	Lyon/Sforzesco	20	7	11	2	1	1
	SpineCor	5	2	2	1	0	0
Dosage of bracing	Full-time	30	12	15	3	1	1
	Intermediate	21	12	9	0	0	0
	Part-time	22	12	9	1	0	0

BMI was lower in failures than non-failures at start of treatment (16.5 ± 2.0 vs 19.1 ± 2.5). Clinical evaluation of kyphosis [33] and major thoracic ATR respectively were also lower in failures at start of treatment (50.0 ± 29.2 vs 68.7 ± 20.5), (10.7 ± 2.9 vs 7.8 ± 3.4). Cobb degrees showed stastically significant lower values in failures, too (32.8 ± 6.3 vs 25.1 ± 6.9). We did not find differences in terms of gender, type and hours of brace prescribed, and type of curve. In terms of treatment, failures showed reduced compliance to bracing (88.2 ± 14.5% vs 93.4 ± 13.0%); moreover, usual physiotherapy or no exercises versus PSE were more frequent in failures than in successes (82% vs 47%).

Finally, successes had statistically significant improvements in all parameters (Table 5); in failures there were no statistically significant differences with treatment (neither improvements or worsening).

**Table 5:** Clinical results

		**Success**			**Failure**		
		**Average**	**Standard deviation**	**P**	**Average**	**Standard deviation**	**P**
Scoliosis curves (Cobb degrees)	Thoracic	-3.89	6.26	0.0001	2.33	17.90	NS
	Thoracolumbar	-7.41	7.22	0.0001	5.00		
	Lumbar	-6.67	6.79	0.0001	-7.00	2.83	NS
	Maximum	-6.32	7.03	0.0001	0.00	15.77	NS
	Main curves	-6.37	7.36	0.0001	0.44	17.57	NS
ATR (degrees)	Thoracic	-2.73	2.34	0.0001	-1.63	4.24	NS
	Thoracolumbar	-3.65	2.84	0.0001	-7.50	3.54	NS
	Lumbar	-3.58	3.05	0.0001	-4.50	0.71	NS
Plumbline distances (mm)	C7	-7.90	11.82	0.0001	0.91	18.82	NS
	T12	-8.15	31.00	0.0500	-0.91	16.25	NS
	L3	-2.98	13.92	NS	0.45	18.77	NS
	C7+L3	-10.89	20.68	0.0001	1.36	27.21	NS
Decompensation (mm)	Lateral	-3.8 7	11.92	0.0500	-5.45	14.40	NS
	Sagittal	3.15	14.29	NS	2.73	13.30	NS
TRACE (points)		-3.02	2.34	0.0001	-1.32	2.77	NS

## Discussion

This cohort prospective study proves the efficacy of a complete conservative treatment of AIS, including bracing and exercises, in the high risk population proposed by the SRS criteria [10]. The Efficacy analysis showed a percentage of fusions of 1.6%, and of progression of 4.8%.

Since 10 patients dropped from the study before ending treatment, the percentage of failures in the Intent-to-treat analysis was 15.1%: even if this is the correct methodological approach, in clinical terms the real possibility that all these patients would have been in the end (or will ever be in their future) surgical is doubtful: in fact their average curvature was very far from the surgical threshold (22.7°; range 16°-34°) with a low risk of progression bone age (Risser 2 in 66.6% of cases). This is the second prospective study respecting the SRS criteria [10]; since the first one was focused on the SpineCor brace [9], this is the first one focusing mainly on rigid braces. A main characteristic of this paper is that it focuses on a general conservative approach to conservative treatment, more than on specific braces. In fact, many different braces have been included, some developed by the authors according to their own correction concepts [21], others by different researchers following various concepts [22,23]. In this respect, the choice of different instruments have been made on an individual basis according to the expertise of the treating physicians.

When compared to other studies in the literature, this prospective paper shows very low surgical rates, comparable to some already presented in the past [9,17,31,34-36], but substantially different from others [18,37]. The main possible explanations of these results include the efficacy of the brace used [2,13,38], the expertise in bracing [11,15], the management of patients possibly able to increase general compliance to treatment [11,15,24]: in the perspective of the conservative experts of SOSORT [11,15] all these points, resumed in the SOSORT criteria [11], should be respected.

Another key point confirmed by this study is the fact that AIS patients adequately treated can improve, and not only avoid progression. This possibility has been already carried forward in previous studies [9,17,25,31,34-36], and should be included as a possible outcome criterion, together with those proposed by the SRS [10].

Moreover it was possible to find some differences at baseline between failures and successes. These include characteristics well known as possibly negative prognostic factors, like low BMI, flat back and severe thoracic curve [15]. Nevertheless, these factors should be regarded very cautiously in this study, since failures mainly included drop-out, i.e. patients that did not complete treatment. Coherent with this point, is the low referred compliance in this group, and the performance of Usual Physiotherapy.

The main strength of this paper is the prospective design coming from a wide clinical database: this allowed to be very selective in the inclusion criteria: consequently the population is very coherent and representative. Moreover, this study represents the everyday clinical reality, and not an experimental set-up: this increases its ecological reliability. The main weaknesses are the observational design, and the absence of a control group: the last one was already considered by the SRS when it defined its inclusion criteria [10], that should represent the population at highest risk of progression. In this respect, this study can be very easily compared to the others with the same design [9,16-19], that in a way are comparable control groups. Some previously published papers strongly support the results of the current study in terms of the effectiveness of bracing, but the comparisons are limited by the lack of a complete respect to the SOSORT and SRS criteria [39,40]. This point of view justify the choice of a design of the study strictly respecting these criteria, which have been proposed only by consensus, but aim to make comparable the results obtained by different group of researchers.

The inclusion of different brace type and the association with exercise can be interpreted as a limitation of the study, because of the effects of confounders. However the scientific literature is not yet able to demonstrate what is the best brace for scoliosis treatment, and it is not yet possible to objectively measure the effects of exercises. In addition the study was done in a everyday clinical setting and not in experimental setting. Another possible limitation is that the treatment management was done by different specialists; according to their personal experience and the specific clinical need of patients. Though all patients included into the study were treated in the same Istitute specialized in the treatment of spine pathologies. All specialists involved have a high grade of experience and work in the same team thus sharing with all the collegues the same strategies for brace treatment. All experts involved into the study strictly follow the actual international guidelines. All these aspect can contribute to a good uniformity. For what the generalizability of the study is concerned, these results can be applied in any super specialized Center on scoliosis conservative management. SOSORT criteria require a good team approach, that should be carefully considered and implemented [11]. Moreover, the SOSORT Guidelines give the indications for personalization of brace treatment according to individual scoliosis patients needs [15]. Finally, the braces used have been well described in the literature [21].

For this study no funding was received, nor there was any conflict of interest issue.

## Conclusions

Bracing in patients with adolescent idiopathic scoliosis who satisfy SRS criteria is effective in reducing progression, and preventing surgery. Combining bracing with exercises according to SOSORT criteria increases treatment efficacy.

## Competing interests

Stefano Negrini has a stock of ISICO (Italian Scientific Spine Institute) of Milan. The other authors do not have any possible conflict of interest.

## Authors’ contributions

All authors participated in data collection and patients’ management and treatment. SN conceived the study, and participated in its design and coordination and drafted the manuscript. FZ participated in the design of the study. SN was responsible for conception and design of the study, analysis of data, and drafted the paper. All the authors contributed in interpreting the data, and revised, read and approved the final paper.

## Authors’ information

All authors are Medical Doctor. SN is a Professor.

## Pre-publication history

The pre-publication history for this paper can be accessed here:

http://www.biomedcentral.com/1471-2474/15/263/prepub
